# The Zebrafish Xenograft Platform—A Novel Tool for Modeling KSHV-Associated Diseases

**DOI:** 10.3390/v12010012

**Published:** 2019-12-20

**Authors:** Eric S. Pringle, Jaime Wertman, Nicole Melong, Andrew J. Coombs, Andrew L. Young, David O’Leary, Chansey Veinotte, Carolyn-Ann Robinson, Michael N. Ha, Graham Dellaire, Todd E. Druley, Craig McCormick, Jason N. Berman

**Affiliations:** 1 Department of Microbiology & Immunology, Dalhousie University, 5850 College Street, Halifax, NS B3H 4R2, Canada; eric.pringle@dal.ca (E.S.P.); chansey.veinotte@Dal.Ca (C.V.); crobinson@dal.ca (C.-A.R.); 2 Beatrice Hunter Cancer Research Institute, 5850 College Street, Halifax, NS B3H 4R2, Canada; dellaire@dal.ca; 3 CHEO Research Institute/Department of Pediatrics, University of Ottawa, Ottawa, ON K1H 8L1, Canada; 4 Department of Pediatrics, Dalhousie University, 5980 University Ave, Halifax, NS B3K 6R8, Canada; ajcoombs13@gmail.com; 5 Division of Hematology and Oncology, Department of Pediatrics, Washington University School of Medicine, St. Louis, MO 63110, USAdavido@wustl.edu (D.O.);; 6 Department of Radiation Oncology, 5820 University Ave, Halifax, NS B3H 1V7, Canada; michael.n.ha@gmail.com; 7 Department of Pathology, Dalhousie University, 5850 College Street, Halifax, NS B3H 4R2, Canada

**Keywords:** Kaposi’s sarcoma-associated herpesvirus (KSHV), human herpesvirus-8, zebrafish, ddPCR, xenotransplantation, primary effusion lymphoma (PEL), hypoxia

## Abstract

Kaposi’s sarcoma associated-herpesvirus (KSHV, also known as human herpesvirus-8) is a gammaherpesvirus that establishes life-long infection in human B lymphocytes. KSHV infection is typically asymptomatic, but immunosuppression can predispose KSHV-infected individuals to primary effusion lymphoma (PEL); a malignancy driven by aberrant proliferation of latently infected B lymphocytes, and supported by pro-inflammatory cytokines and angiogenic factors produced by cells that succumb to lytic viral replication. Here, we report the development of the first *in vivo* model for a virally induced lymphoma in zebrafish, whereby KSHV-infected PEL tumor cells engraft and proliferate in the yolk sac of zebrafish larvae. Using a PEL cell line engineered to produce the viral lytic switch protein RTA in the presence of doxycycline, we demonstrate drug-inducible reactivation from KSHV latency *in vivo*, which enabled real-time observation and evaluation of latent and lytic phases of KSHV infection. In addition, we developed a sensitive droplet digital PCR method to monitor latent and lytic viral gene expression and host cell gene expression in xenografts. The zebrafish yolk sac is not well vascularized, and by using fluorogenic assays, we confirmed that this site provides a hypoxic environment that may mimic the microenvironment of some human tumors. We found that PEL cell proliferation in xenografts was dependent on the host hypoxia-dependent translation initiation factor, eukaryotic initiation factor 4E2 (eIF4E2). This demonstrates that the zebrafish yolk sac is a functionally hypoxic environment, and xenografted cells must switch to dedicated hypoxic gene expression machinery to survive and proliferate. The establishment of the PEL xenograft model enables future studies that exploit the innate advantages of the zebrafish as a model for genetic and pharmacologic screens.

## 1. Introduction

Kaposi’s sarcoma-associated herpesvirus (KSHV, also known as human herpesvirus-8, or HHV8) is the infectious cause of Kaposi’s sarcoma (KS), primary effusion lymphoma (PEL), and multicentric Castleman’s Disease (MCD) [1]. KSHV is a gammaherpesvirus that achieves life-long infection of human hosts by establishing latency in immature B lymphocytes and promoting differentiation into a plasmablast-like cell type [2]. An essential feature of herpesvirus latency is reversibility, and periodic reactivation from latency enables lytic KSHV replication and spread to new hosts. Accordingly, KSHV latency is unstable *in vivo* and *in vitro*, with spontaneous expression of lytic antigens [3]. PEL is a rare disease that occurs most frequently in human immunodeficiency virus (HIV)-positive individuals, or otherwise immunosuppressed individuals. PEL prevalence remains unclear, but a single 15-year institution study concluded that PEL accounts for approximately 4% of non-Hodgkin’s lymphomas (NHLs) [4]. PEL develops as bloody effusions in body cavities, including pleural, peritoneal, and pericardial spaces, but can also form solid extracavity lymphomas [5]. Survival is poor, and the rarity of the disease has contributed to a dearth of clinical trials evaluating the most effective treatments. The current standard of care is EPOCH (Etoposide, Prednisone, Oncovin/vincristine, Cyclophosphamide, Hydroxydaunorubicin/doxorubicin) or CHOP with or without antiretroviral therapy [5].

Patient-derived PEL cell lines can be grown in culture and retain the KSHV episome as a latent infection. While readily amenable to experimentation, these *in vitro* cultures do not fully recapitulate all features of the cancer, thus providing motivation for the development of *in vivo* PEL models. PEL cell lines readily engraft and proliferate in the abdominal cavity of severe-combined immunodeficiency (SCID) mice, or form subcutaneous solid tumors when injected with Matrigel; the latter of which mimics some aspects of the tumor microenvironment by providing an extracellular matrix [6]. In mice, PEL xenografts regress with rapamycin treatment [7], as they do in KS [8], due to the loss of mechanistic target of rapamycin complex 1 (mTORC1)-dependent paracrine and autocrine cytokine signaling required for PEL proliferation [7]. This reliance on paracrine and autocrine signals provides ample rationale for further development of *in vivo* PEL models that afford opportunities to evaluate the influence of the tumor microenvironment.

Zebrafish larvae have emerged as a robust and efficient *in vivo* model for human tumor xenotransplantation (XT), especially human lymphomas and leukemias [9,10,11]. Zebrafish share remarkable genetic similarity with humans and have several advantages as a low-cost experimental model, including high fecundity and rapid development. Zebrafish larvae are optically transparent and lack an adaptive immune system until 28 days post-fertilization [11,12], making them an attractive animal XT model, with no requirement for immunosuppression. Furthermore, the zebrafish XT platform allows for the rapid and direct observation and imaging of tumor-cell dynamics in a live animal microenvironment in real time. Particularly important for blood cancers, the developmental process of hematopoiesis is highly conserved in zebrafish, making it an excellent model to study normal and abnormal blood development and disorders [13,14]. Previously, we successfully transplanted and measured proliferation and migration of leukemia cell lines and primary leukemic cells in zebrafish embryos [9,11,15]. This zebrafish patient-derived xenograft (PDX) platform enables rapid evaluation of patient-tumor-cell response to several anticancer drugs. For example, xenografts from a patient with T-cell acute lymphoblastic leukemia (ALL) harboring a previously uncharacterized *NOTCH1* mutation (A1696D) were specifically susceptible to a gamma secretase inhibitor [11,16]. The success of the zebrafish XT platform for studies using leukemia cells suggests that zebrafish larvae might provide a suitable host environment for PEL and could be utilized for further preclinical drug studies or potentially facilitate rapid patient-derived xenotransplation to inform personalized treatment decisions.

In this study, we successfully engrafted and observed the proliferation of a KSHV-infected PEL cell line and KSHV-infected epithelial cells in zebrafish larvae. We demonstrated that tetracycline (Tet)-inducible gene expression was feasible in the zebrafish XT context, although KSHV reactivation from latency was inefficient in this model. We further demonstrated the sensitivity and specificity of droplet digital PCR (ddPCR) to selectively measure the expression of human and viral genes in xenografted larvae. To assess oxygen levels in the zebrafish larvae, we used a hypoxia-sensitive dye to label cells and confirmed that the yolk sac is a low-oxygen environment. To further explore the effects of the hypoxic microenvironment in the larvae, we silenced expression of eIF4E2, the essential cap-binding protein of hypoxia-specific translation initiation machinery, and demonstrated its requirement for PEL proliferation in the yolk sac. We demonstrated for the first time that viral lymphomas can proliferate in the zebrafish yolk sac in a manner similar to other hematological cancers. Thus, future drug discovery studies aimed at treatments for PEL and other viral lymphomas could similarly benefit from further “*in-Danio*” xenotransplantation approaches.

## 2. Materials and Methods

### 2.1. Ethics Statement and Zebrafish Husbandry

Adult *casper* [13,17] zebrafish were maintained in a recirculating commercial housing system (Pentair Aquatic Eco-Systems, Apopka, FL, USA) at 28 °C in 14 h:10 h light:dark conditions and bred according to standard protocol [15,18]. Embryos were collected and grown in E3 medium (5 mM of NaCl, 0.17 mM of KCl, 0.33 mM of CaCl_2_, and 0.33 mM of MgSO_4_) in 10 cm Petri plates at 28 °C. Embryos were cleaned and provided with new media every 24 h and used experimentally before 7 days post-fertilization (dpf). Zebrafish embryos (0–72 h post-fertilization) are considered to enter the larval stage after 3 days post-fertilization (dpf). The use of zebrafish in this study was approved by and conducted in accordance with the policies of the Dalhousie University Committee on Laboratory Animals, under protocols #17-055 (approved 1 May 2017) and #17-131 (approved 1 January 2018).

### 2.2. Cell Lines

Body-cavity-based lymphoma (BCBL1) cells are a clone derived by limiting dilution of patient-derived PEL cells [16,19]. TREx-BCBL1-RTA cells are subclone of BCBL-1 engineered to express the KSHV immediate early gene *RTA* under the control of a tetracycline promoter [17,20]. Both cell lines were cultured in suspension with RPMI-1640 supplemented with 10% *v*/*v* fetal bovine serum (FBS) (Thermo Fisher Scientific, Grand Island, NY, USA), 100 IU of penicillin and streptomycin (Life Technologies Inc., Burlington, ON, Canada) and 55 µM beta-mercaptoethanol (Life Technologies Inc., Burlington, ON, Canada). The iSLK.219 cells are a subclone of a Caki-1 derived epithelial cell line engineered to express RTA under a tetracycline promoter [18,21]. The iSLK.219 cells were latently infected with the recombinant rKSHV.219 virus [9,19]. The iSLK.219 cells and 293T cells used for lentivirus generation were maintained in DMEM supplemented with 10% *v*/*v* FBS and 100 IU of penicillin and streptomycin. rKSHV.219 has a puromycin resistance cassette, and retention of the viral episome in culture required supplementation with 10 µg/mL puromycin (Life Technologies Inc., Burlington, ON, Canada) [9,20]. All cells were maintained at 37 °C in a 5% CO_2_ atmosphere. For *in vitro* growth curves, cells were washed with phosphate-buffered saline (PBS) (Wisent, Inc., St-Bruno, QC, Canada) and seeded at 2.5 × 10^5^ cells/mL. Live cells were counted by using Trypan Blue (Life Technologies Inc., Burlington, ON, Canada) and a hemocytometer. To enumerate red fluorescent protein (RFP)+ iSLK.219 cells, cells were fixed with 4% paraformaldehyde for 15 min at room temperature, and nuclei were stained with Hoechst 33342 (Life Technologies Inc., Burlington, ON, Canada). Fluorescent images were captured with an EVOS FL Cell Imaging system (Thermo Fisher Scientific, Grand Island, NY, USA) and RFP+ and Hoeschst+ cells were counted, using a custom CellProfiler version 3.0.0 script [9,21].

### 2.3. Zebrafish Xenotransplantation

Approximately 5 × 10^6^ BCBL cells or TREx-BCBL1-RTA cells were harvested and centrifuged at 1000 × *g* for 5 min. Approximately 2 × 10^6^ iSLK.219 cells were dissociated from culture dishes with trypsin and recovered in full media before pelleting. Cell pellets were resuspended with 10 mL of PBS and 5 µg/mL of CellTracker Orange CMTMR Dye (Life Technologies Inc., Burlington, ON, Canada). Cells were incubated at 37 °C for 20 min and then collected by centrifugation. Cells were washed twice with 10 mL of cell culture medium and once with 10 mL of PBS. Cells were resuspended to a final volume of approximately 100–150 µL in culture medium for injection. CMTMR Dye was omitted on xenografting of iSLK.219 cells to test in vivo reactivation. To test for oxygen concentration in the yolk sac, TREx-BCBL1-RTA cells were incubated with 5 µM of Image-iT Green Hypoxia Reagent (Life Technologies Inc., Burlington, ON, Canada) for 30 min at 37 °C. Cells were washed once with PBS after labeling.

Zebrafish embryos were allowed to naturally shed their chorion at 2 dpf. Before injection, embryos were anesthetized with 0.09 mg/mL of tricaine solution (Sigma-Aldrich Canada Co., Oakville, ON, Canada) and arrayed on an agarose plate for cell transplantation, as described previously [9]. Experimental cells were loaded into a pulled-glass capillary needle and allowed to settle in the bore of the needle. The needle was then attached to a PLI-100A Pico-Liter microinjection system (Harvard Apparatus, Holliston, MA, USA), and yolk sacs were manually injected with 50–100 cells. The following day, a fluorescent Discovery V20 stereomicroscope (Carl Zeiss Canada Ltd., North York, ON, Canada) was used to screen for larvae with an obvious bolus of fluorescent cells in the yolk sac. Following injections, larvae were kept at 35 °C for the remainder of the experiment, an established midpoint between the optimal temperature for zebrafish development (28 °C) and human cell growth (37 °C) [9,13].

### 2.4. Zebrafish Xenotransplant Ex Vivo Cell Proliferation Assay

Positive larvae (those with a compact bolus of cells present in the yolk sac) were separated into appropriate experimental groups of 30–40 and monitored daily in 60 × 15 mm plates at 35 °C. For XT cell-proliferation data, cells were quantified ex vivo at 24 h post-injection (hpi) (baseline) and 72 hpi (experimental endpoint). Twenty larvae were euthanized by tricaine overdose (1 mg/mL) and dissociated in collagenase (Sigma-Aldrich Canada Co., Oakville, ON, Canada) for 30 min. Once dissociated into a single-cell suspension, 200 µL of FBS was added to the sample to slow the enzymatic reaction prior to collagenase removal. The sample was then centrifuged at 300 × *g* for 5 min, the supernatant was removed, and the sample was washed once with a 30% *v*/*v* FBS in PBS solution and centrifuged once more. The supernatant was removed, leaving the fluorescently labeled human tumor cells among the zebrafish cells. The sample was resuspended in 10 µL/larva solution of 30% *v*/*v* FBS in PBS for imaging. Ten 10 µL boli were pipetted onto a “PTFE” printed glass slide with a 5 mm well diameter (Electron Microscopy Sciences, Hatfield, PA, USA) and allowed to settle for 8–10 min. The boli were individually imaged using an inverted Axio Observer Z1 microscope (Carl Zeiss Canada Ltd., North York, ON, Canada) and the images were analyzed using ImageJ software (NIH) as described previously [9,22]. Cell numbers for each experimental group were normalized to baseline cell counts to ensure that cells were engrafting and proliferating in the XT model. Experiments were conducted in triplicate for each cell line. Any remaining larvae were euthanized with tricane overdose prior to 7 dpf, the limit that the Dalhousie University Committee on Laboratory Animals has approved for these studies in protocols #17-055 (approved May 1^st^, 2017) and #17-131 (approved January 1^st^, 2018).

### 2.5. Zebrafish Toxicity Experiments

To determine an appropriate doxycycline treatment dose for zebrafish larvae, toxicity assays were conducted to determine the half-maximum tolerated dose (MTD_50_) [9,19]. *Casper* larvae [13,20] staged at 72 h post-fertilization (hpf) were arrayed in 96-well plates and treated with increasing doses of drug for a total of 72 h to ascertain toxicities. Treatment doses for experiments were chosen by halving the dose when 80% survival was observed. Toxicity assays were repeated in triplicate.

### 2.6. Western Blotting

TREx-BCBL1-RTA cells were harvested by centrifugation at 1500 × *g* for 5 min, washing once with ice-cold PBS, pelleting again, and then lysing in 2 × Laemmli buffer (4% *w*/*v* sodium dodecyl sulfate (SDS), and 20% *v*/*v* glycerol, 120 mM of Tris-HCl pH 6.8). Samples were reduced with 100 mM of dithiothreitol (DTT) and boiled at 95 °C for 5 min. An aliquot of the lysate before reduction and boiling was used to determine the protein concentration using the DC Protein-Assay (Bio-Rad Laboratories (Canada) Ltd., Mississauga, ON, Canada). Concentrations were normalized, and 10 µg of total protein content was analysed by SDS-PAGE and transferred to PVDF membranes, using a semi-dry transfer (Trans-Blot Turbo Transfer System and RTA PVDF kit, Bio-Rad). Membranes were blocked with 5% *w*/*v* BSA TBS-T and then probed overnight with the following antibodies: myc (New England Biolabs Canada Ltd., Whitby, ON, Canada (NEB) #2276); ORF57 (Santa Cruz Biotechnology, Inc., Santa Cruz, CA, USA; sc135746); ORF65 (a kind gift from Jae Jung); β-actin (NEB #5125); eIF4E2 (Cedarlane, Burlington, ON, Canada; GTX103977); and eIF4E1 (NEB #2067). Primary antibody binding was detected with horseradish–peroxidase conjugated secondary antibodies (anti-mouse: NEB #7076; anti-rabbit #7074). Blots were developed with Clarity–ECL chemiluminescence reagent and imaged on a Chemidoc-Touch (Bio-Rad Laboratories (Canada) Ltd., Mississauga, ON, Canada).

### 2.7. RT-qPCR Analysis

RNA was harvested from TREx-BCBL1-RTA cells, using RNeasy (Qiagen, Inc., Toronto, ON, Canada), according to the manufacturer’s directions. Cells were harvestd by centrifugation at 1500× *g* for 5 min and lysed in RLT buffer from the RNeasy kit. The cDNA was generated by using Maxima H Minus First Strand Reverse Transcriptase (Life Technologies Inc., Burlington, ON, Canada) with random oligo priming and qPCR performed, using GoTaq (Fisher Scientific, Ottawa, ON, Canada) on a CFX Connect Realtime PCR system (Bio-Rad Laboratories (Canada) Ltd., Mississauga, ON, Canada) using the following primers (5′–3′): ORF45 (Forward (F): TGA TGA AAT CGA GTG GGC GG, Reverse (R): CTT AAG CCG CAA AGC AGT GG), K8.1 (F: AGA TAC GTC TGC CTC TGG GT, R: AAA GTC ACG TGG GAG GTC AC), β-actin (F: CTT CCA GCA GAT GTG GAT CA, R: AAA GCC ATG CCA ATC TCA TC), RTA (F: GAT TAC TGC GAC AAC GGT GC, R: TCT GCG ACA AAA CAT GCA GC), 18S rRNA (F: TTC GAA CGT CTG CCC TAT CAA, R: GAT GTG GTA GCC GTT TCT CAG G). An annealing temperature of 60 °C was used for all primer pairs. The abundance of a transcript *x* was normalized to 18S rRNA abundance, using the following formula:

Abundance = 2^(−ΔCq)^

where ΔCq = Cq_*x*_ − Cq_18S_, and Cq is the quantitative cycle, as determined automatically by the CFX Manager software (Bio-Rad Laboratories (Canada) Ltd., Mississauga, ON, Canada).

### 2.8. RNA Extraction and Digital Droplet PCR (ddPCR)

Twenty XT larvae were euthanized and transferred to a 1.5 mL microcentrifuge tube. Water was carefully removed, and larvae were subsequently lysed in Buffer RLT (Qiagen RNeasy Plus kit) supplemented with 40 mM DTT. The larvae were homogenized by repeated passage through a 22-gauge needle (at least 20 times). RNA isolation was conducted according to the manufacturer’s recommended protocol, including an on-column DNAse digestion (Qiagen, Inc., Toronto, ON, Canada). Eluted RNA was quantified by spectrophotometry on a GE Nanovue instrument (GE Healthcare, Mississauga, ON, Canada) and concentrations were equalized prior to reverse transcription with Maxima H (Life Technologies Inc., Burlington, ON, Canada) with random oligo priming, as described above. The cDNA solution was diluted 1:10 for subsequent ddPCR analysis on the Bio-Rad QX200 ddPCR platform. Then, 20 µL reaction mixtures were assembled, using 2 × QX200 ddPCR EvaGreen supermix (Bio-Rad Laboratories (Canada) Ltd., Mississauga, ON, Canada), 5 µL of diluted cDNA, and 200 nM of each forward and reverse primer (same primers sequences as described above for RT-qPCR analysis). We included both an RT negative control for cDNA generation and no template controls during PCR, to exclude genomic DNA or carryover amplicon contamination. Droplets were generated, and PCR was conducted according to the manufacturer’s instructions using a 60 °C annealing temperature. Fluorescent intensity of droplets were analyzed using QuantaSoft software (Bio-Rad Laboratories (Canada) Ltd., Mississauga, ON, Canada).

### 2.9. Lentivirus Generation

eIF4E2 was silenced using pGIPZ shRNA-expressing lentivirus (ThermoFisher clone V2LHS_68041) or a non-targeting control (clone RHS_4346). Lentiviruses were generated by co-transfecting pGIPZ with psPAX2 and pMD2.G packaging plasmids (kind gifts from Didier Trono via Addgene, Watertown, MA, USA; #12259, #12260) in HEK293T cells with polyethylenimine MAX (Polysciences, Inc., Warrington, PA, USA). Two days after transfection, virus-containing cell supernatants were harvested and cleared with a 0.45 µm filter. Virions were aliquoted and stored at –80 °C, prior to transduction. We transduced suspension cells by diluting the suspension culture in an equal proportion with lentivirus stock in the presence of 4 µg/mL of polybrene (hexadimethrine bromide) (Sigma-Aldrich Canada Co., Oakville, ON, Canada) and incubating for 24 h. Inoculum was then removed by centrifugation, and cells were cultured for several days, in the presence of 1 µg/mL of puromycin (Life Technologies Inc., Burlington, ON, Canada) until a consistently GFP+ and puromycin resistant culture was obtained.

### 2.10. Statistics and Data Processing

Numerical data were collected and organized in Excel (Microsoft), and histograms were generated in Prism (GraphPad Software, San Diego, CA, USA). All statistical tests were calculatd in Prism: * = *p* < 0.05, ** = *p* < 0.01, *** = *p* < 0.001 and ns = non-significant. Error bars are standard error measurement (SEM).

## 3. Results

### 3.1. KSHV-Infected PEL Cells Successfully Engraft in Zebrafish Embryos

**Figure 1:**
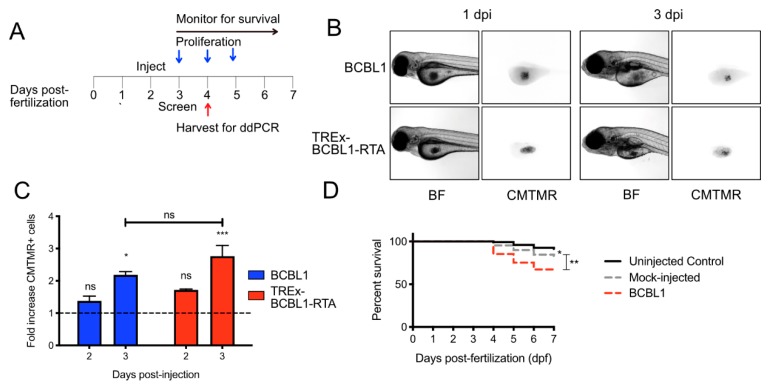
Proliferation of BCBL and TREx-BCBL1-RTA in zebrafish larvae: (**A**) timeline of xenotransplantation experiment. Fish were xenotransplanted with fluorescent CMTMR-labeled, primary effusion lymphoma cells by microinjection at 2 days post-fertilization (dpf). The following day, embryos were visually screened with a fluorescent microscope for viability and the presence of a cell bolus in the yolk sac. Groups of larvae were sacrificed at indicated times for dissociation and counting of xenotransplanted cells or RNA harvest. Survival of the larvae was monitored throughout the experiment; (**B**) photomicrographs of xenotransplanted larvae demonstrating the bolus of cells in the yolk sac at 1 and 3 days post-injection (dpi), which are 3 and 5 dpf, respectively; (**C**) proliferation of BCBL1 and TREx-BCBL1-RTA cells at 2 and 3 dpi normalized to the number of cells counted at 1 dpi (*n* = 3 independent experiments with cells from 20 larvae counted per measurement; means ± SEM; statistical significance was determined by two-way ANOVA compared to the cell counts at 1 dpf); (**D**) CMTMR-labeled BCBL1 cells were injected into 2 dpf embryos, which were screened at 3 dpi. Then survival was monitored until 7 dpf. Uninjected and media mock-injected embryos were included as controls (*n* = 150 larvae per group accrued from 3 separate hatchings; statistical significance was determined by Mantel-Cox test; * = *p* < 0.05, ** = *p* < 0.01, *** = *p* < 0.001).

To determine whether KSHV infected lymphoma cells can successfully engraft in zebrafish, we labeled BCBL1 cells, or the TREx-BCBL1-RTA subclone with Tet-regulated reactivation, with the cell-permeable dye CMTMR, and microinjected them into the yolk sac of 48 h post-fertilization (hpf) zebrafish embryos. The dye remains incorporated in cells over multiple cycles of cell division, with the signal diminishing proportionally after each division. The following day, these larvae were visually screened for boli of fluorescent cells in the yolk sac, and groups of fish were dissociated every day, for three days, after initial screening (Figure 1A). The cells appeared to remain in the yolk sac, and we did not detect evidence of migration of tumor cells into surrounding tissues (Figure 1B). Both BCBL1 cells and TREx-BCBL1-RTA cells successfully proliferated in the yolk sac, with BCBL1 cells increasing in number by 2.2-fold, and TREx-BCBL1-RTA cells increasing by 2.8 fold over three days (Figure 1C). We also monitored the impact of the injection process itself on larval survival. Embryos were either injected at 2 dpf, with cells labeled with CMTMR or were “mock-injected”, whereby sterile cell culture media was injected into the yolk sac. Mock-injected larvae had a slight yet significant decrease in survival compared to uninjected controls by five days post-injection (i.e., 7 dpf). Xenografted larvae had significantly poorer survival at 7 dpf compared to mock-injected controls (Figure 1D). These data indicate that PEL cells can successfully proliferate in the yolk sac and that the larvae can tolerate xenotransplantation; however, the microinjection process diminishes the long-term viability of the larvae.

### 3.2. KSHV-Infected Epithelial Cells Successfully Engraft and Can Be Induced for Lytic Replication

**Figure 2:**
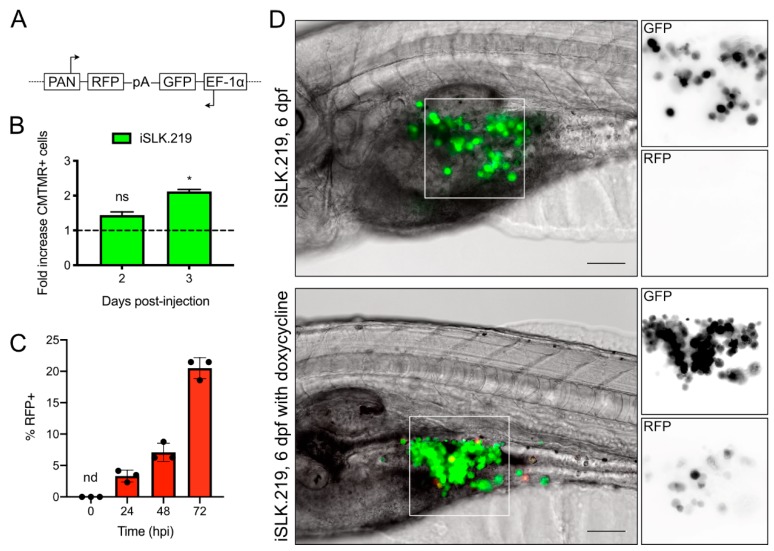
Proliferation and lytic reactivation of iSLK.219 in zebrafish embryos: (**A**) simplified diagram of rKSHV.219 reporter construct, adapted from [17,19]. Latently infected cells express GFP from a constitutive EF-1α promoter. During lytic replication, the immediate early protein RTA binds to the viral PAN promoter and stimulates RFP expression. A polyA (pA) signal sequence is present on both strands of the viral genome; (**B**) proliferation of iSLK.219 cells at 2 and 3 dpi, normalized to the number of cells counted at 1 dpi (*n* = 3 independent experiments with cells from 20 larvae counted per measurement; means ± SEM; statistical significance was determined by two-way ANOVA compared to the cell counts at 1 dpf; * = *p* < 0.05); (**C**) iSLK.219 cells were treated with 1 µg/mL of doxycycline and fixed at the times indicated, or left untreated (Time = 0 hpi). Cells were fixed with 4% paraformaldehyde, and nuclei were stained with Hoescht. RFP+ cells and nuclei were imaged on an inverted fluorescent microscope and enumerated with CellProfiler (*n* = 3 independent experiments ± SD; nd = not detected). (**D**) iSLK.219 were injected into the yolk sac of 2 dpf zebrafish embryos. The following days, larvae were screened for viability and a GFP+ cell bolus by fluorescence microscopy. In half of the larvae, the E3 media was supplemented with 40 µg/mL doxycycline, which was refreshed daily. Xenotransplanted larvae were monitored daily for RFP+ cells. Presented here are representative images of both doxycycline-activated and mock-treated larvae at the ethical endpoint of the experiment. We could observe RFP+ cells in the yolk sac of approximately 20% of larvae treated with doxycycline, and none in untreated larvae (scale bar = 100 µm).

KSHV establishes a latent infection in most epithelial cell lines, and all PEL cell lines are latently infected with KSHV [19,22]. Reactivation from latency requires expression of the immediate early gene *regulator of transcriptional activation (RTA)* that initiates an ordered cascade of gene expression to subvert the host, replicate the viral genome, and package genomes into virions. Furthermore, iSLK.219 cells can be stimulated to induce lytic replication through Tet-driven expression of RTA. We wanted to determine if we could stimulate viral gene expression in zebrafish xenografts by adding doxycycline directly to the embryo water. We pursued these experiments by using iSLK.219 cells that were latently infected with the rKSHV.219 virus. These cells constitutively express green fluorescent protein (GFP) from an EF-1α promoter on the viral episome. Reactivation from latency is visualized by accumulation of red fluorescent protein (RFP) reporter driven by the viral PAN promoter (Figure 2A) [9,19]. We first tested whether these KSHV-infected epithelial cells would proliferate within the yolk sac. We injected 2 dpf *casper* embryos with iSLK.219 cells, and similar to what was found with BCBL cells, iSLK.219 cells readily proliferated in the yolk sac (Figure 2B). The bright GFP fluorescence of the iSLK.219 cells allowed for clear detection of the cell bolus using standard GFP filter sets (500–550 nm), despite the high autofluorescent background of the embryos. This allowed us to omit CMTMR Dye from reactivation experiments and readily detect reactivated RFP+ cells.

In cell culture, iSLK.219 cells maintain tight control over latency, and few RFP+ cells can be detected [20,23]. The addition of doxycycline stimulates the immediate early *RTA* transgene, and within 24 h, RFP+ cells can be detected in culture, the proportion of which increases over time (Figure 2C). We attempted to reactivate iSLK.219 cells *in vivo* by adding doxycycline directly to the water in which the larvae were immersed. We tested the toxicity of a range of doxycycline concentrations (0–180 µM) and found that ~80% of fish could tolerate a 100 µM dose. We then used 40 µg/mL doxycycline for our fish treatment. One day after injection, 3 dpf embryos containing a GFP+ cell bolus were isolated and treated with 40 µg/mL doxycycline, which was refreshed daily. Similar to what we observed in cell culture (Figure 2C) we were unable to detect RFP+ cells in boli of xenotransplanted iSLK.219 cells, even after four days in the yolk sac (Figure 2D). In the doxycycline-treated fish, we could observe some RFP+ cells after three days of treatment (6 dpf). However, this process was inefficient, and we could only observe RFP+ cells in ~20% of the larvae, and very few cells within each larvae were detectably RFP+ (Figure 2D). While inefficient, this suggests that it is possible to activate a Tet-responsive promoter in xenotransplanted cells in the yolk sac and stimulate viral gene expression *in vivo*.

### 3.3. Droplet Digital PCR (ddPCR) Can Detect Human and Viral Transcripts in a PEL Xenograft

Monitoring xenografted cells in the zebrafish typically relies on prior fluorescent labeling of the cells. In this study and in others, our group employed fluorescence or human antibody immunohistochemistry-based ex vivo quantification, to enumerate xenografted cells at various time points [9,24]. Techniques to measure changes in gene expression in human xenografts have been hindered by the paucity of human transcripts in the background of zebrafish RNA. In this study, we took advantage of the sensitivity and specificity of droplet digital PCR (ddPCR), to measure changes in gene expression in our xenografts [23,25]. In ddPCR, the PCR solution is emulsified into droplet suspension to partition the cDNA into positive or negative reactions that are recorded through a microfluidic fluorescence detector.

**Figure 3:**
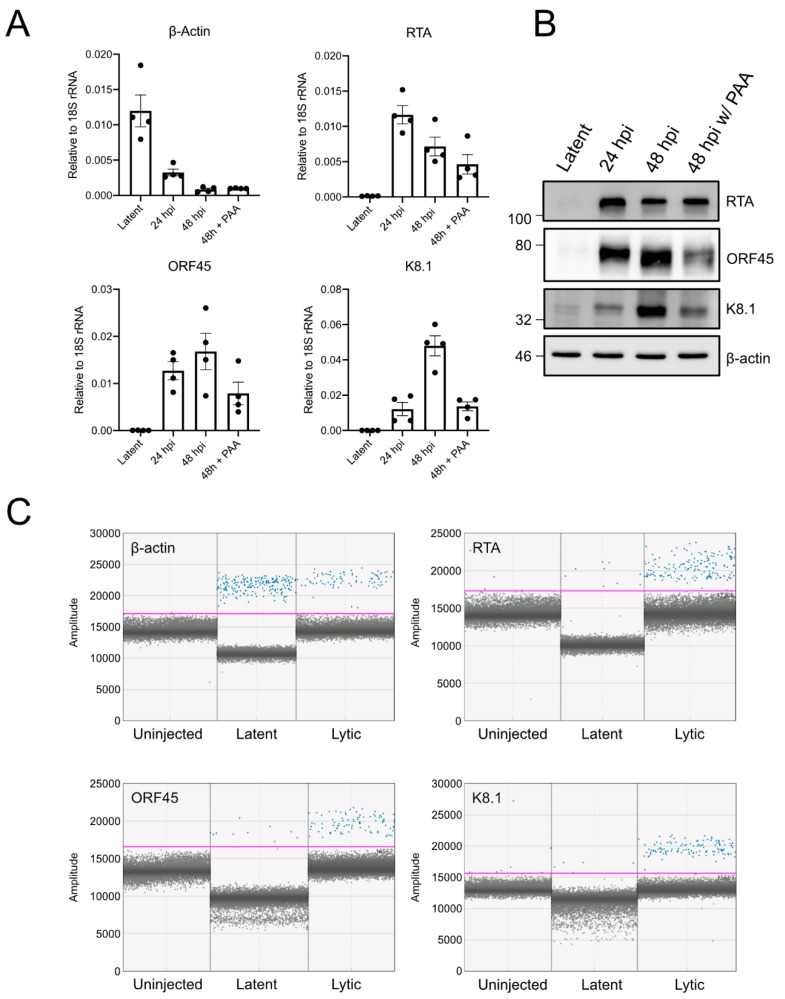
Detection of viral gene expression in xenotransplanted cells by ddPCR: (**A**) TREx-BCBL1-RTA cells reactivated with 1 µg/mL of doxycycline in culture and RNA was harvested at latent cells, or cells undergoing lytic replication at 24 or 48 h post-induction (hpi). Then, 500 µM phosphonoacetic acid (PAA) was used to inhibit replication of the viral genome and late gene expression; RT-qPCR was used to measure transcript abundance of β-actin, *RTA* (immediate early), *ORF45* (early) and *K8.1* (late) (*n* = 4 independent experiments; means ± SEM); (**B**) Western blot of cells treated as in (A) to confirm accumulation of target proteins; (**C**) ddPCR amplification plot for β-actin, *RTA, ORF45,* and *K8.1*. The *x* axis displays individual events, and the *y* axis is fluorescence amplitude. For all targets, we tested cDNA generated from uninjected larvae, or larvae injected with untreated TREx-BCBL1-*RTA* cells or cells treated with 1 µg/mL doxycycline for 12 h prior to injection. RNA was harvested from larvae at 48 hpi. The pink threshold line separates positive reaction droplets (blue) from negative droplets (gray).

The full KSHV lytic gene expression program is initiated by the immediate early gene product RTA, which stimulates expression of early genes that subvert the host cell, counter innate immune defences, and initiate viral genome replication. Late gene expression follows viral genome replication and generates structural proteins required for virion assembly and genome packaging. Like the iSLK.219 cells, TREx-BCBL1-RTA cells express RTA under a Tet-inducible promoter [17,26]. Doxycycline treatment reactivates the virus from latency; early gene expression (e.g., ORF45) can be detected after 24 h, and late gene expression (e.g., K8.1) can be readily detected by 48 h post-reactivation. Late gene expression is dependent on genome replication, which can be prevented by using phosphonoacetic acid (PAA), a herpesvirus DNA-polymerase inhibitor [24,27] (Figure 3A,B).

Due to the inefficiency of stimulating reactivation from latency in xenotransplanted iSLK.219 cells, we instead pretreated the TREx-BCBL1-RTA cells with doxycycline for 12 h to initiate reactivation from latency prior to injection into the yolk sac. We then harvested total RNA from the xenotransplanted larvae at 2 dpi (Figure 3C). We were able to detect human *β-actin* (*ACTB*) transcripts in the injected larvae but not in our mock-injected controls, demonstrating that it is possible to detect human mRNAs from a limited number of xenotransplanted cells against the background of far more abundant zebrafish transcripts. We were also able to detect the KSHV immediate early transcript *RTA*, the early transcript *ORF45*, and the late transcript *K8.1* in doxycycline-treated xenotransplanted cells. We could also detect *RTA* and *ORF45* in cells that were not treated with doxycycline, possibly in response to the yolk sac microenvironment or simply representing spontaneous entry into lytic replication. Because KSHV late gene expression is licensed by viral genome replication, the detection of late *K8.1* transcript in xenografts indicates that the larval yolk sac microenvironment supports viral genome replication and late gene expression.

### 3.4. Engraftment of PEL Cells in the Yolk Sac Requires the Hypoxic Translation Initiation Factor eIF4E2

**Figure 4:**
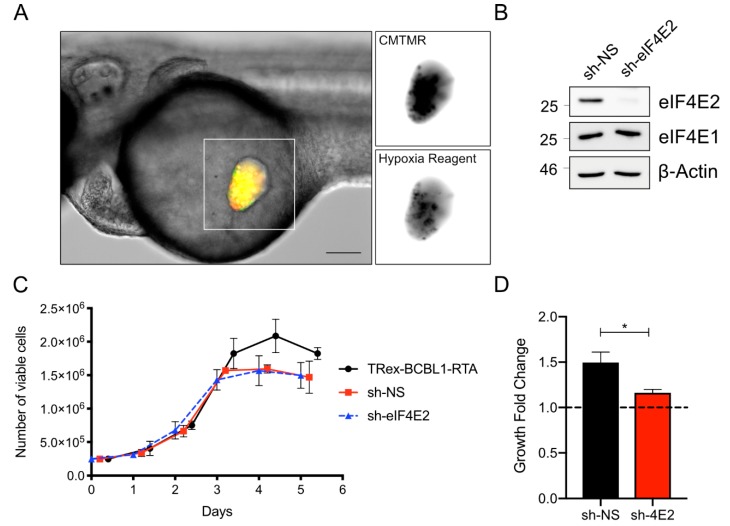
The zebrafish yolk sac is hypoxic and xenotransplant proliferation requires eIF4E2: (**A**) TREx-BCBL1-RTA cells were labeled with 1 µM of Image-iT Green Hypoxia Reagent for 30 min prior to washing and labeling with CMTMR dye. Cells were injected into 2 dpf embryos and imaged 1 hour later. Scale bar = 100 µm. (**B**) TREx-BCBL1-RTA cells were transduced with eIF4E2 shRNA or a non-targeting control lentivirus. Cells were harvested and probed for eIF4E2 and homologue eIF4E1 by Western blotting; (**C**) TREx-BCBL1-RTA cells or cells transduced as in (**B**) were seeded at 2.5 × 10^5^ cells/mL and monitored for viability and proliferation by manual counting, using a hemocytometer and trypan blue for the following five days (*n* = 3 independent transductions; means ± SEM; statistical significance was determined by two-way ANOVA); (**D**) proliferation of transduced TREx-BCBL1-RTA cells at 3 dpi normalized to the number of cells counted at 1 dpi (*n* = 3 independent experiments with cells from 20 larvae counted per measurement; means ± SEM; statistical significance was determined by two-way ANOVA compared to the cell counts at 1 dpf; * = *p*-value < 0.05).

The zebrafish yolk sac provides a suitable environment for proliferation of many human cancer cells, including KSHV-infected cells, as we have demonstrated (Figure 1 and Figure 2). However, the yolk sac differs from the typical cancer microenvironment because it is acellular, non-vascularized, lacks an extracellular matrix, and is rich in lipids [25,27]; as such, successful proliferation of xenotransplanted cells likely requires metabolic adaptation to this environment. Despite the yolk sac being a common site for xenotransplantation in the zebrafish, little is known about the challenges that a cancer cell encounters in this environment that may affect the interpretation of experiments. Thus, it is important to characterize this microenvironment to better understand the cellular requirements of xenotransplantation and to identify factors, such as oxygen concentration, that could interfere with expected drug activities [26,27]. Although the tissue surrounding the yolk sac is vascularized, we hypothesized that the yolk sac itself might be a relatively hypoxic environment. We employed the cell-permeable dye Image-iT Green Hypoxia Reagent that only fluoresces at oxygen concentrations below 5% to measure of the oxygen concentration in the zebrafish yolk sac. Specifically, we labeled TREx-BCBL1-RTA cells with this dye, followed by CMTMR, and injected 2 dpf embryos, as described above. We monitored the embryos after injection, and, within an hour, we could observe the appearance of many green fluorescent cells, suggesting they were experiencing a low-oxygen environment (Figure 4A).

Given these qualitative results indicating that the yolk provides a hypoxic environment, we sought to determine if the xenografted cells themselves where responding to hypoxia. To address this possibility, we examined the role of the eIF4F complex in xenografted cell proliferation. In normoxia, the canonical eIF4F complex governs global translation initiation [26,27]. In hypoxia (<5% oxygen) eIF4F is disassembled due to mTORC1 inactivation, and the hypoxic eIF4F initiation factor (eIF4F_H_) assumes a primary role in promoting translation initiation [10,27]. This complex is activated by the stabilization of hypoxia inducibility factor 2α (HIF-2α) during hypoxia and its subsequent binding to the m^7^GTP cap binding protein eIF4E2 [12,27]. eIF4F_H_ is required for protein synthesis in hypoxia and cancer cell proliferation in the hypoxic core of a solid tumor in nude-mice xenografts or in spheroid culture [9,27]. Consequently, we hypothesized that xenografts experiencing low-oxygen conditions require eIF4F_H_ activation to proliferate.

To investigate the importance of hypoxic protein synthesis in TREx-BCBL-RTA cells in the zebrafish yolk sac, we silenced eIF4E2 expression by transducing cells with lentiviruses bearing eIF4E2-specific shRNA; lentiviruses bearing a non-targeting shRNA served as a negative control for this experiment. We observed efficient eIF4E2 silencing, with no discernable off-target silencing of the eIF4F component eIF4E1 (Figure 4B). In normal cell-culture conditions in atmospheric oxygen, there was no difference in proliferation between either the non-targeting shRNA control or the eIF4E2 shRNA compared to parental TREx-BCBL1-RTA cells (Figure 4C). However, when these cells were xenotransplanted into the yolk sac, the eIF4E2-silenced cells failed to proliferate as readily as the control cells (Figure 4D). Taken together, these experiments indicate that the zebrafish yolk sac is a functionally hypoxic environment that requires metabolic compensation by the xenografted cells to proliferate.

## 4. Discussion

Zebrafish have been used to model a variety of hematopatholocial malignancies [10,28]. Because zebrafish exclusively rely on innate immunity until adaptive immunity develops at approximately 28 dpf, human cells can engraft and proliferate in zebrafish larvae without the need for immunosuppression [12,29]. Building upon our expertise with zebrafish XT models of leukemias and lymphomas, we developed the first *in vivo* model for a viral lymphoma in zebrafish. We demonstrated that KSHV-infected PEL cells proliferate in the embryo yolk sac. The embryos tolerated xenotransplantation of the cells, with animal survival rates comparable to mock-injected controls. We took advantage of the well-established paradigm of herpesvirus latent–lytic switch to determine whether we could successfully activate a Tet-regulated cassette in a xenograft by adding doxycycline directly to the fish water. While we were able to demonstrate Tet-driven expression of the *RTA* lytic switch gene *in vivo* by monitoring RFP expression in xenografted iSLK.219 cells, this was relatively inefficient. It is possible that the density of cells in the yolk sac could limit lytic reactivation through RTA-dependent activation of the *NOTCH1* pathway that suppresses lytic gene expression in neighbouring cells [30]. To our knowledge, this is the first demonstration that Tet-regulated promoters in xenotransplanted cells can respond to doxycycline in the fish water, which may be useful in other studies requiring post-XT stimulation of gene expression.

Zebrafish xenografts are typically evaluated by measuring XT cell proliferation and migration into other tissues, and determining whether exogenous chemicals can impact these processes [9,28]. There are few studies of gene expression in the xenografted cells, as there are so few transplanted cells compared to the zebrafish cells that XT transcripts cannot be easily detected at a quantitative threshold by using conventional RT-qPCR methodology [28]. In this study, we developed sensitive new methods to detect viral and host gene expression in xenotransplants using ddPCR. The ease and sensitivity of ddPCR technology suggests that it may also be adapted to replace current laborious microscopy-based methods for monitoring xenograft cell proliferation. The relatively low quantity of XT human transcripts compared to abundant larval host transcripts has made it difficult to quantify differences in abundance of XT transcripts. This limitation can be surmounted by probing abundant Alu repeat retroelements by qPCR; there are ~10^6^ Alu copies per human genome [15,29]. While preparing this article, Salo and colleagues reported that RT-qPCR can be used to monitor XT cell proliferation by targeting the abundant glyceraldehyde-3-phosphate dehydrogenase (GAPDH) transcript [28,31]. They also demonstrated that the low-abundance cytokeratin 17 transcript could be detected by ddPCR and that the number of detected copies/µL tightly correlated with the size of the XT, as determined by both fluorescent quantification and qPCR for GAPDH [28,32]. We suspect that the sensitivity of ddPCR is likely sufficient to directly quantify XT load by detection of XT DNA. Nevertheless, we maintain that the primary application of ddPCR technology in zebrafish XT models will likely be gene-expression analysis of XTs in response to drug treatments or changes in the XT microenvironment.

We took advantage of established methods for Tet-regulated control of KSHV latent-lytic switch, to determine whether we could stimulate gene expression in the xenograft and if we could detect viral gene expression by using the specific and highly sensitive ddPCR platform. We successfully detected mRNA from all temporal classes of gene expression following dox treatment, and we observed moderate lytic gene expression in untreated cells. Even though we could detect KSHV lytic gene products in the xenograft, it remains unclear whether this is sufficient to support production of infectious virions *in situ*, which will require further development of sensitive detection methods. It has been reported that the normal maintainence temperature of zebrafish (28 °C) can hinder replication of human viruses that have evolved to replicate at higher temperatures [15,33]. We have addressed this issue by housing xenotransplanted zebrafish larvae at 35 °C to enable KSHV replication. Another human herpesvirus, herpes simplex virus type 1, has been shown to replicate in nervous tissue of adult zebrafish housed at 28 °C [31]. However, since KSHV is highly restricted to primates and cannot productively infect other mammals, including mice [32], we reasoned that zebrafish cells would be unlikely to support KSHV replication. Anecdotally, we did not notice any GFP+ cells distal to the XT-injection site that could indicate dissemination of xenografted human cells or KSHV infection of zebrafish cells, and we did not attempt to directly infect the zebrafish embryos or zebrafish cell lines with KSHV in this study.

A recent study conducted by our group suggested that the yolk sac likely does not provide the same extracellular matrix context present in a solid tumor [33]. As a result of this microenvironment feature, resistance to anoikis, a mode of programmed cell death initiated after loss of contact with the extracellular matrix, is required to support increased XT proliferation in this compartment. Here, we demonstrated that xenografts likely also experience a hypoxic environment in the yolk sac, with an oxygen tension below normal tissue “physoxia” of 5%, and more similar to many solid tumors [34,35]. This microenvironment requires specific metabolic compensation by activation of the eIF4E2-dependent eIF4F_H_ translation initiation complex in the xenograft. Hypoxia drives significant changes in both the transcriptome and the proteome. However, most of the changes to the proteome are derived from a global reprogramming of the translational efficiencies of mRNA rather than changes to the transcriptome [36]. In future studies, the hypoxic state of the yolk sac should be considered when modeling human cancers in zebrafish embryos.

Hypoxia can influence the proliferation and migration of cancer cells, partially as a result of the influence of oxygen concentration and hypoxia-inducible factors on angiogenesis [37,38]. Furthermore, the responses of cancer cells to multiple drugs can be altered in hypoxic conditions. For example, hypoxia can induce resistance to cisplatin treatment in multiple cancer types [39,40]. This is an important factor to consider when designing zebrafish XT experiments targeting the yolk sac, especially in a drug-screening schema, where the effects of compounds may be masked or amplified as a result of low oxygen levels. Embryos can tolerate xenotransplantation in other anatomical sites, such as the circulation, the hindbrain ventricle, or the perivitelline space, which should be considered to be injection sites, along with the yolk sac, in the design of XT studies [10].

KSHV latency is unstable in culture, and lytic gene expression can be detected in a small percentage of cells. Accordingly, the presence of lytic gene products RTA and ORF45 in non-doxycycline treated xenotransplants could reflect normal rates of spontaneous lytic replication, or the virus could be responding to the hypoxic environment of the yolk sac. Several hypoxia response elements (HREs) have been detected in the KSHV genome, most notably including HREs adjacent to the *RTA* lytic switch gene [41,42,43]. *RTA* expression during hypoxia is sufficient to stimulate lytic replication, but only a small percentage of cells reactivate in these conditions [41]. Remarkably, the transcriptome of KSHV latently infected cells resembles a hypoxic gene expression signature, possibly due to the actions of the latent protein LANA that increases levels HIF-1α mRNA and protein [44], suggesting that HRE activity in infected cells could be influenced by products of the latent transcriptome, as well as by environmental oxygen. Later during lytic replication, the ORF34 lytic protein binds and stabilizes HIF-2α [45], which could promote eIF4E2-dependent translation [27]. Interestingly, HIF-1α is required for normal lytic gene expression during normoxia in both KSHV and murine gammaherpesvirus 68 infections [46,47]. In our experiments, the differential effects of eIF4E2 silencing on PEL proliferation suggests that KSHV latency does not fully recapitulate a hypoxic phenotype and suggests that KSHV selectively accesses the hypoxic gene expression program.

In summary, we present a novel zebrafish xenograft model for PEL as a convenient low-cost alternative to existing murine models that obviates the need for immunosuppressive treatments. This model enables evaluation of the role of hypoxia in both KSHV and other cancer cells in a complex 3D microenvironment. Further study will be required to understand how metabolic compensation allows cancer cells to proliferate in this niche. This model could serve as an excellent platform for patient-derived xenograft (PDX) experiments, akin to those done by others [36]. However, unlike murine models or immune-deficient adult zebrafish models [38], these experiments only require small numbers of cells, conserving primary patient material that could be in low supply. Nonetheless, it is important to note that these larval experiments will not replace murine or adult zebrafish xenograft models; rather, it is our hope that this model can supplement the battery of techniques already available to study PEL and other viral cancers *in vivo*.
